# Application of Volatilome Analysis to the Diagnosis of Mycobacteria Infection in Livestock

**DOI:** 10.3389/fvets.2021.635155

**Published:** 2021-05-24

**Authors:** Pablo Rodríguez-Hernández, Vicente Rodríguez-Estévez, Lourdes Arce, Jaime Gómez-Laguna

**Affiliations:** ^1^Department of Animal Production, International Agrifood Campus of Excellence (ceiA3), University of Córdoba, Córdoba, Spain; ^2^Department of Analytical Chemistry, Inst Univ Invest Quim Fina and Nanoquim Inst Univ Invest Quim Fina and Nanoquim (IUNAN), International Agrifood Campus of Excellence (ceiA3), University of Córdoba, Córdoba, Spain; ^3^Department of Anatomy and Comparative Pathology and Toxicology, International Agrifood Campus of Excellence (ceiA3), University of Córdoba, Córdoba, Spain

**Keywords:** diagnosis, livestock, mycobacteria, volatilome, veterinary

## Abstract

Volatile organic compounds (VOCs) are small molecular mass metabolites which compose the volatilome, whose analysis has been widely employed in different areas. This innovative approach has emerged in research as a diagnostic alternative to different diseases in human and veterinary medicine, which still present constraints regarding analytical and diagnostic sensitivity. Such is the case of the infection by mycobacteria responsible for tuberculosis and paratuberculosis in livestock. Although eradication and control programs have been partly managed with success in many countries worldwide, the often low sensitivity of the current diagnostic techniques against *Mycobacterium bovis* (as well as other mycobacteria from *Mycobacterium tuberculosis* complex) and *Mycobacterium avium* subsp. *paratuberculosis* together with other hurdles such as low mycobacteria loads in samples, a tedious process of microbiological culture, inhibition by many variables, or intermittent shedding of the mycobacteria highlight the importance of evaluating new techniques that open different options and complement the diagnostic paradigm. In this sense, volatilome analysis stands as a potential option because it fulfills part of the mycobacterial diagnosis requirements. The aim of the present review is to compile the information related to the diagnosis of tuberculosis and paratuberculosis in livestock through the analysis of VOCs by using different biological matrices. The analytical techniques used for the evaluation of VOCs are discussed focusing on the advantages and drawbacks offered compared with the routine diagnostic tools. In addition, the differences described in the literature among *in vivo* and *in vitro* assays, natural and experimental infections, and the use of specific VOCs (targeted analysis) and complete VOC pattern (non-targeted analysis) are highlighted. This review emphasizes how this methodology could be useful in the problematic diagnosis of tuberculosis and paratuberculosis in livestock and poses challenges to be addressed in future research.

## Introduction

Analysis of volatile organic compounds (VOCs) is an emerging research area in both human and veterinary medicine (1), which allows a non-invasive, fast, and economic diagnosis as well as identification of new biomarkers as alternative to current diagnostic techniques (2). VOCs are defined as a sub-category of small molecular mass substances within metabolites, which are characterized by its low boiling point and high vapor pressure (3, 4). VOCs are produced into the environment, allowing a direct measuring in the gas phase and offering a minimum sample handling, a non-invasive monitoring, and an easier sampling compared with other metabolites which have to be extracted from biological samples (5, 6). In this context, volatilome (or volatome) is the VOCs' signature produced by an organism (7–10).

The volatilome has a wide variety of uses and applications, such as diagnosis of infectious diseases (11) and neoplasia (12), distinction between vaccinated and non-vaccinated animals (13), monitoring of antibiotic treatment (14), differentiation of diet composition (15, 16), and even evaluation of reproductive parameters (17). Because VOCs are constantly emitted during metabolic processes, the detection of VOC profiles might enable the development of novel non-invasive diagnostic tools (7).

The identification of VOCs produced by pathogens, host–pathogen interactions, and biochemical pathways, either associated with homeostasis or pathophysiological responses, has become the volatilome into an approach of growing interest for the diagnosis of infectious diseases (18). Pathologic processes have the capacity to modify VOCs' patterns either by producing new volatile substances or by the metabolic consumption of VOC substrates that are normally present (19). Consequently, the diagnostic approach of VOC analysis provides two perspectives, the search of new biomarkers and the identification of biomarkers lost along a pathological process (1).

Infection of livestock by slow-growing mycobacteria, such as those grouped under *Mycobacterium tuberculosis* complex (MTBC), especially *Mycobacterium bovis*, as well as *M. avium* subsp. *paratuberculosis* (MAP), might take advantage of the development of faster and sensitive diagnostic techniques. Considering the growth requirements of these mycobacteria, as well as other factors associated with the host immune response after infection, diagnosis of mycobacterial infections becomes a challenge, especially in the livestock sector. The diagnosis of the infection by mycobacteria is currently based on different tedious, expensive, laborious, and time-consuming methodologies (20–22). Thus, the analysis of VOCs could be proposed as an innovative strategy to improve the diagnostic field of these infections (Table 1) supported by the fact that, historically, people suffering from tuberculosis had a characteristic breath smell (24). The research carried out in this context has used different biological matrices, such as serum (33, 34), breath (34, 35), feces (13, 28), and microbiological culture (36–39) to identify biomarkers related to diseases produced by mycobacteria.

**Table 1:** *In vivo* studies evaluating VOC analysis as a diagnostic tool for mycobacterial infection in animals.

**Mycobacteria species and animal species**	**Matrix**	**Analytical technique**	**Kind of infection**	**Sensitivity**	**Specificity**	**References**
***Mycobacterium bovis***						
Cattle	Serum	EN	Experimental	–	–	(23)
Badger	Serum	EN	Natural and experimental	–	–	(23)
Badger	Serum	SIFT-MS	Natural	88%	62%	(24)
Cattle	Exhaled breath	GC-MS EN	Natural	– 100%	– 79%	(25)
Cattle	Exhaled breath	ATD-GC-MS	Experimental	–	–	(26)
Cattle	Exhaled breath	GC-MS	Experimental	83.8–96.4%	97.4–99.2%	(18)
Cattle	Serum	EN	Natural	–	–	(27)
White-tailed deer	Feces	GC-MS	Experimental	78.6%	91.4%	(13)
Cattle	Feces	GC-MS	Experimental	83–100%	100%	(28)
Wild boar	Exhaled breath	GC-MS	Natural	100%	90%	(29)
Wild boar	Feces	GC-MS	Natural	100%	80%	(29)
***Mycobacterium avium*** **subsp**. ***paratuberculosis***						
Cattle	Serum	EN	Natural	–	–	(30)
Goat	Exhaled breath and feces	DMS	Experimental	–	–	(1)
Goat	Exhaled breath and feces	GC-MS	Experimental	–	–	(31)
Goat	Exhaled breath and feces	GC-MS	Experimental	Exhaled breath: 90.3% Feces: 86.6%	Exhaled breath: 81.8% Feces: 85.0%	(32)

*ATD-GC-MS, thermal desorption–gas chromatography–mass spectrometry; DMS, differential mobility spectrometry; EN, electronic nose; GC-MS, gas chromatography–mass spectrometry; GC-GC-MS, two-dimensional gas chromatography–mass spectrometry; SIFT-MS, selected ion flow tube mass spectrometry*.

Although the use of VOCs obtained from different biological samples to diagnose diseases is considered as a big hope with a promising future, now it remains at a developing stage (40). One of the main hurdles against the development of this new strategy is the lack of standardization between studies which often leads to non-comparable results (40, 41). Few detailed *in vivo* studies are available on the analysis of VOCs as a diagnostic tool for mycobacterial infection in animals. In light of these premises, the present review collects the available literature from the volatilome approach to evaluate the recent methodologies and procedures used as an attempt of improvement of the diagnosis of infection by mycobacteria in livestock, focusing on the infection by MTBC (*Mycobacterium bovis*) and MAP, to point out future research lines of interest to be implemented.

## Mycobacteria Target of Study

Mycobacteria belong to the genus *Mycobacterium* which includes the MTBC, with all the causative species of human and mammal tuberculosis; the *M. avium* complex (MAC), which also comprises species of relevance in human and veterinary medicine, such as MAP; as well as environmental rapid and slow-growing non-tuberculous mycobacteria. These all are aerobic and immobile bacilli with specific growing conditions which include pathogenic, opportunistic, and saprophytic species (42, 43). While there are many species, such as *M. tuberculosis* and *M. bovis*, known for being the etiological agents of important human and animal diseases, rapid- and slow-growing non-tuberculous mycobacteria used to be minority species, which should be considered because of their interference with the currently established diagnostic strategies (44).

### *Mycobacterium tuberculosis* Complex (*Mycobacterium bovis*)

MTBC is composed by a broad group of mycobacteria species characterized for its genetic proximity and its pathogenic ability of affecting humans, such as *M. tuberculosis* and *M. africanum*, and a wide variety of wild and domestic animals, such as *M. bovis* and *M. caprae*. *M. bovis* stands out for being the primary etiological agent responsible for bovine tuberculosis (bTB), also considered as the main cause of animal tuberculosis due to the multi-host character of this bacterium (45). Animal tuberculosis is a zoonotic disease with great impact on public health, agriculture, wildlife, and trade areas (20, 46). In this sense, although most cases reported as human tuberculosis are caused by *M. tuberculosis*, ~30% of these cases are related to *M. bovis* infection (zoonotic tuberculosis) (28), especially in developing countries (47) where prevalence of livestock bTB becomes substantial (48–50). Despite huge efforts that are currently focused on the eradication of bTB, there are many difficulties mainly associated with the performance of the different diagnostic techniques as well as with other geographical and epidemiological conditions, which make it very difficult in endemic countries (51). Therefore, zoonotic tuberculosis is often under-reported, emphasizing the importance of providing appropriate diagnostic tools in livestock to reach the eradication of *M. bovis* and reduce zoonotic tuberculosis cases.

### *Mycobacterium avium* subsp. *paratuberculosis*

MAP is the causative agent of paratuberculosis (PTB) or Johne's disease, a chronic infection that affects the small intestine of ruminants resulting in a marked reduction of animal productivity (31) and sometimes in death (1). MAP is also believed to be related to Crohn's disease, a chronic bowel disease in humans, although this fact is yet to be defined (52–54).

The main importance of PTB comes from the great economic losses in animals due to reduced milk and meat yields as well as slaughter value (32). MAP diagnosis becomes a challenge because of its pathogenesis: while the main clinical signs are only present in the late progression of the disease, when the body condition is severely affected, the animals intermittently spread bacteria during a previous subclinical phase. These features result in a low sensitivity of the current direct (fecal culture and genome detection) and indirect (specific antibodies detection) diagnostic methods (55, 56) (Table 2). Hence, reliable and complementary diagnostic methodologies are of key importance to enhance the current diagnostic repertoire of techniques focused on the identification of infected animals so as to improve the sensitivity of the diagnosis.

**Table 2:** Sensitivity and specificity parameters from conventional diagnostic techniques.

**Technique**	**Sensitivity**	**Specificity**	**References**
***Mycobacterium bovis***			
**Intradermal skin test**			
*Caudal fold test*	68–96.8%	96–98.8%	(46)
*Cervical intradermal test*	80–91%	75.5–96.8%	(46)
*Comparative cervical test*	55.1–93.5%	88.8–100%	(46)
**Gamma interferon evaluation**	74.00% 73–100%	≥99% 80–90%	(57) (46)
**Microbiological culture**	72.9–82.8%	97.1–100.0%	(58)
**Serologic assays—ELISA**			
*From milk samples (MPB70+MPB83)*	50.0%	97.5%	(59)^‡^
*From sera samples (recombinant antigen cocktails)*	40.6–93.1%	69.7–99.1%	(60)^‡^
*From sera or plasma samples (recombinant antigen cocktails)*	62.7–69.5%	97.2–98%	(IDEXX)^†^, ^‡^
**PCR**			
*IS6110*	82.5–92.3%	94.3–99.0%	(58)
*MPB70*	94.59%	96.03%	(61)
***Mycobacterium avium*** **subsp**. ***paratuberculosis***			
**Microbiological culture**	23–70%	100%	(62)
**Serologic assays—ELISA**	7–94%	40–100%	(62)
**PCR**			
*IS900*	79.3–91.0%	88.3–93.9%	(63)

† *Mycobacterium bovis antibody test kit, IDEXX Laboratories Inc*.

‡ *ELISA results compared with culture or single intradermal comparative cervical test positive and negative status*.

## Routine Diagnostic Techniques Against MTBC and *M. avium* subsp. *paratuberculosis*

The diagnosis of mycobacterial infection is currently at the center of attention because, although well-established and reliable, it has its own limitations. Apart from being a tedious process, special consideration must be given to the lack of an optimal diagnostic sensitivity (Table 2) and the different variables which may interfere with the methods and techniques in use (2, 21). Therefore, an accurate and reliable diagnostic methodology of the infection by mycobacteria, or a combination of various strategies, is the cornerstone of their control (23).

### Current *Ante-mortem* and *Post-mortem* Diagnostic Techniques Against MTBC

Field and *ante-mortem* surveillance tests against MTBC infection are mainly based on the detection of a delayed-type hypersensitivity response to the intradermal skin test (IST) through the inoculation of purified protein derivative from *M. bovis* (bPPD; tuberculin protein), and on quantifying the concentration of gamma interferon (IFN-γ) after culturing blood samples in the presence of tuberculin, in the case of IFN-γ assay test, a supplemental or confirmatory test (25). IST is the OIE prescribed test for international trade and is currently considered as the official diagnostic screening technique in many countries worldwide; it is the primary *ante-mortem* test to support control and eradication programs in different geographical areas (46), responsible for its effectiveness as it is compulsory in the slaughtering of those animals with a positive result (64). In Europe, the aforementioned information is regulated by the Council Directive 64/432/EEC. Although IST and IFN-γ assay have reasonable sensitivity and good specificity (Table 2), both techniques require a minimum of 48–72 h to obtain a result (21, 65) besides presenting other disadvantages and limitations. On the one hand, IST requires visiting the farm and restraint of the animals twice, and a delicate and difficult administration and interpretation of skin results, which may vary due to differences in tuberculin doses, site of application (Table 2), and interpretation schemes (25, 27, 66); on the other hand, IFN-γ assay implies a complex laboratory methodology (18), a considerably more expensive price than a skin test (65, 67), and suffers from cross-reactivity with other related mycobacteria resulting in false-positive results (2). In addition, performance of these tests can be compromised by factors associated with the immune response and health status of the animal leading to a misinterpretation of the results. Development and use of a pre-screening test before field tests would be useful to reduce work efforts and diagnostic time (27).

Although microbiological culture is considered the gold-standard approach for the diagnosis of mycobacterial infection, it is characterized by a long incubation time to confirm the presence of mycobacteria (around 8–12 weeks) (2). Furthermore, the isolation of mycobacteria sometimes requires specific compounds such as mycobactin, a siderophore which determines the viability and growth of some mycobacteria species, as is the case of MAP; and, sometimes, additional steps such as decontamination. For all these reasons, culture becomes a tedious and laborious, although necessary, option in *M. bovis* diagnosis.

Other *in vitro* assays, such as serologic assays (ELISA) or PCR, have limitations associated with accuracy and execution that restrict their use (66). While ELISA sensitivity is affected by the delayed and irregular antibodies response in bTB (68), PCR is considered a postmortem diagnostic option with promising findings but still under development, focused on the search of markers that ensure diagnostic sensitivity (61) (Table 2). Therefore, the reliability of these tests depends on the stage of infection and, in addition, these require transporting of animal samples to the laboratory, which finally increases diagnostic time too (40), highlighting the interest on the availability of portable equipment.

### Current *Ante-mortem* and *Post-mortem* Diagnostic Techniques Against *M. avium* subsp. *paratuberculosis*

The intermittent and sometimes low shedding of the mycobacteria as well as the irregular seroconversion in the subclinical phase of PTB (69, 70) gives a limited sensitivity to the *in vivo* diagnosis (32), currently based on serological assays (ELISA) and PCR from feces. Although ELISA has a limited sensitivity (Table 2), the irregular spread of bacteria *via* feces has raised serology as the most common technique used for the monitoring of PTB (71) due to its cheap and easy use. In addition, fecal shedding and immune response vary individually to a large extent (72). For example, the sensitivity of PCR methods can be affected by the variable bacterial load in samples and the co-purification of PCR inhibitors during DNA extraction (73). Therefore, and although the current combination between serology, vaccination (when regulation allows this option due to its possible interference on bTB eradication campaigns), and slaughtering constitutes a strategy with remarkable effectiveness, there is a need for diagnostic tests with higher sensitivity and decreased processing time to reduce false-negative results and enable effective disease control strategies, as different authors have highlighted before (31, 32). Volatilome evaluation has been capable of discriminating MAP infection before clinical illness occurs, offering an early diagnosis and significant time savings (1). This could be considered as one of the main advantages against the current techniques in use.

In short, against the current situation, it would be of help to have an *ante-mortem* diagnostic methodology capable of detecting mycobacterial infection with repeatability, a good quality/price ratio, high sensitivity and specificity, and rapid detection and obtaining of results. VOC strategy could be a complementary option because it mostly fulfills these requirements, and it has been successfully used for mycobacterial diagnosis in many animal species (Table 3). In this sense, an initial approximation analyzing stable air as matrix has been proposed to evaluate MAP infection in cattle (74); however, due to the low number of infected animals included within each infected group, further studies are required to confirm the suitability of this approach.

**Table 3:** VOCs related to mycobacterial infection in different animal species (targeted analysis).

**Mycobacteria species**	**Potential discriminatory compounds or type of compounds**	**Animal species**	**Matrix**	**Analytical technique**	**References**
*Mycobacterium bovis*	2,3-Dimethyl, 1,3- pentadiene 1,3-Dimethylbutyl cyclohexane	Cattle	Exhaled breath	GC-MS	(25)
*Mycobacterium bovis*	>100 compounds (acetone, dimethyl sulfide, and 2-butanone as the most abundant)	Cattle	Exhaled breath	ATD-GC-MS	(26)
*Mycobacterium bovis*	4-Hydroxy-4-methyl-2-pentanone Benzaldehyde 1-Ethyl-2-pyrrolidinone α, α-Dimethyl-benzenemethanol **Nonanal**	Cattle (1 year old)	Exhaled breath	GC-MS	(18)
*Mycobacterium bovis*	Methylbenzene **Hexanal** 2-Methyl pyridine 2,4-Dimethyl pyridine 2-(1,1-Dimethoxy)-ethanol^†^ **2-Ethyl-1-hexanol** Benzene acetaldehyde 3,7-Dimethyl-6-octenyl-(2E)-2-butanoate Acetophenone^†^ 4-Methyl-phenol 2-Decanone^†^ (–)-Beta-fenchol 1-Decanol **Indole** 3-(1,1-Dimethylethyl)-4-methoxy-phenol 1-Octadecanol 2-Dodecanone	White-tailed deer (12–18 months old)	Feces	GC-MS	(13)
*Mycobacterium bovis*	Thioether Thiophene Aldehyde Organosulfur (sulfone) Imine Pyridine derivative Amino acid Ketone Alcohol **Indole** Diterpenoid alkane Fatty acyl (amino acid derivative) Diterpene alcohol Dicarboxylic acid and derivative	Cattle (120–121 days old)	Feces	GC-MS	(28)
*Mycobacterium bovis*	Adult animals (>2 years): O-Cymene Juvenile animals (<12 months): Acetic acid, methyl ester **3-Methylpentane** Trichloromethane α-Methylstyrene Decane 4,6,8-Trimethyl-1-nonene 1,3-Bis(1,1-dimethylethyl)-benzene 2,5-Dimethylhexane-2,5-dihydroperoxide 2,5-Bis(1,1-dimethylethyl)-phenol Heptacosane 5-Butyl-5-ethylheptadecane 11-Decyl-tetracosane 11-(1-Ethylpropyl)-heneicosane 3-Ethyl-5-(2-ethylbutyl)-octadecane	Wild boar (juveniles, sub-adults, adults)	Exhaled breath	GC-MS	(29)
*Mycobacterium bovis*	Sub-adult animals (12–24 months): 10,18-Bisnorabieta-8,11,13-triene Juvenile animals (<12 months):	Wild boar (juveniles, sub-adults, adults)	Feces	GC-MS	(29)
	**Acetone** Toluene 2,6-Bis(1,1-dimethylethyl)-4-(1-methylpropyl)- phenol				
*Mycobacterium avium* subsp. *paratuberculosis*	1-Propanol **2-Butanone** **Acetone** Benzene 2-Methyl-butanal Ethylbenzene **Hexanal** **Nonanal** Styrene	Goat (21–55 weeks old)	Exhaled breath	GC-MS	(31)
*Mycobacterium avium* subsp. *paratuberculosis*	Pentane Hexane Heptane **Acetone** **2-Butanone** 2-Pentanone 2-Hexanone 2-Heptanone 3-Octanone 3-Methyl-2-butanone 3-Methyl-2-pentatone Methyl isobutyl ketone Isoprene **Methyl acetate** Dimethyl sulfide Dimethyl disulfide Furan 2-Ethylfuran 2-Methylfuran **3-Methylfuran** 2-Pentylfuran	Goat (21–55 weeks old)	Feces	GC-MS	(31)
*Mycobacterium avium* subsp. *paratuberculosis*	45 compounds. Top-3 (random-forest): **3-Methylfuran** 2,3-Butanedione **Methyl acetate**	Goat (2–3 weeks old)	Feces	GC-MS	(32)
*Mycobacterium avium* subsp. *paratuberculosis*	51 compounds. Top-3 (random-forest): **3-Methylpentane** **2-Ethyl-1-hexanol** 2-Methylpentane	Goat (2–3 weeks old)	Exhaled breath	GC-MS	(32)

*ATD-GC-MS, thermal desorption–gas chromatography–mass spectrometry; GC-MS, gas chromatography–mass spectrometry; GC-GC-MS, two-dimensional gas chromatography–mass spectrometry*.

† *Statistically significant trends identified for vaccinated and infected animals but not in non-vaccinated and infected animals*.

*Consistent compounds between different assays are highlighted in bold*.

## Impact of the Experimental Setting on the VOCS Profile

### *In vitro* vs. *in vivo* Studies

Analysis of VOCs as a diagnostic option for mycobacterial disease has been evaluated both *in vivo* and *in vitro*. Compared with *in vivo* assays, the large number of existing *in vitro* studies, which basically consist in mycobacteria culturing, reveals the early stage of development where this research area stands (36–38, 75, 76). The reviewed literature in the present study suggests some drawbacks related to those *in vitro* studies.

First, mycobacteria growth, which as aforementioned requires several weeks or even months, is required to identify changes in the analysis of VOCs from microbiological culture to allow the distinction between negative and positive samples. In other words, although *in vitro* experiments can detect VOC changes related to different stages of the mycobacteria growth (76, 77), it still takes a long time to identify these changes, which is one of the main disadvantages linked to the current diagnostic methodology. Accordingly, researchers point to reduce the diagnostic time by avoiding the limiting step of culturing and suggesting other innovative techniques such as VOC measurement directly *in vivo* (32).

It is also important to highlight the low correlation existing between results obtained from cultured bacteria compared with those VOCs produced from other biological samples studied in *in vivo* experiments (40). For example, (32) detected two compounds only present above MAP cultures which were ranked among the top discriminating VOCs in their statistical analysis. However, in the comparison with their *in vivo* results, these two compounds tended to be in lower concentration in MAP-inoculated animals compared with non-inoculated animals. This situation emphasizes the caution required when adopting *in vitro* findings to *in vivo* conditions since the influence from the host, its microbiome and host–microbiome interactions (78), as well as the influence from environmental factors, such as diet, age, or drug use (40), needs to be considered. In addition, another hurdle of the *in vitro* settings is related to the different VOC profiles obtained depending on the substrate where the mycobacteria grow resulting in inconclusive findings (79).

The effectiveness of *in vivo* approach is supported by the results of many studies where VOCs from biological samples have been used to distinguish between infected animals with different mycobacteria species and non-infected animals (1, 18, 31, 80). Many different biological matrices such as serum, breath, or feces have been studied as a source of information for VOC analysis in this field, existing great differences between their nature and characteristics. This constitutes another problem in the comparison between *in vitro* vs. *in vivo* experiments, giving inconsistent results. When (31) compared *in vivo* results obtained from feces and breath samples with the *in vitro* VOC profiles obtained from different MAP strains' culture by (77), their conclusions were not very clarifying: from more than 100 substances detected in feces and breath, only 15 and 5 of them, respectively, were found in the bacterial *in vitro* pattern.

### Experimental vs. Natural Infection

Another variable to consider when evaluating VOCs as an option for mycobacterial diagnosis in animals is the type of infection: natural or experimental. Although experimental infections are logically the most common and easy option for this kind of approximation, studies in naturally infected animals are of paramount importance. Experimental infections allow controlling different environmental conditions that may impact on the results, being the most studied option in the analysis of VOCs for mycobacterial diagnosis (Table 1). However, assays with natural infections are needed to validate the results obtained from any new diagnostic tool, such as volatilome analysis, in experimental settings. Along this review, only a single article has been found to include the analysis of VOCs from both experimentally and naturally infected animals (23). These researchers found that differences between negative and positive animals were more pronounced in the natural infection group than in the experimentally infected one. This fact highlights the importance of performing studies in field conditions in the future to compare with those with experimentally infected animals and to validate the results from the latter ones.

## Species Under Study

The analysis of VOCs has been used in many species for the diagnosis of mycobacterial infection. Livestock species are the most frequent ones, probably because of the importance and repercussion of bTB and PTB for farm animals. As expected, bovine is the most studied animal model with this innovative approach, followed by goats (Table 1). Our findings are consistent with the wide variety of diseases that have been tested through this methodology in cattle, such as bovine respiratory disease (11), mastitis (81), brucellosis (30), ketosis (82), or ketoacidosis (83, 84).

Remarkably, diagnosis through volatilome has been also performed in wildlife, more specifically in deer (13), badger (23, 24), and recently in wild boar (29), with much effort put into the development of a better disease surveillance methodology on these species. Among laboratory animals, although outside the scope of this review, non-human primates have been used to study the mycobacteria species which usually affect humans, *M. tuberculosis* (80, 85), as well as the murine model, which has been also employed to assess the use of breath for mycobacterial infection (41).

The encouraging results obtained in these studies with different animal species highlight the great potential of this methodology in MTBC and MAP diagnosis. However, there is a lack of studies in other species of interest, such as the pig, an animal model with an increasing interest in biomedical research (86). Furthermore, the marked differences that exist among different animal species make feasible that different approaches may be necessary for each species. This review highlights the starting point where this new diagnostic approach stands and the necessity of further studies and research before its setting up as an alternative routine or field technique.

## Biological Matrices

VOCs can be detected directly from different biological samples such as blood, serum, breath, feces, sweat, skin, urine, or vaginal fluids (13, 27, 87–89), opening up huge opportunities for this new diagnostic methodology. Although samples should be initially selected according to the disease and the pathogenesis of the agent, there are multiple options that allow collection of alternative samples. For example, the predominantly respiratory character of bTB would place exhaled breath as the most appropriate sample to study this disease. However, there are studies that show interesting results for the analysis of VOCs from *M. bovis*–infected animals using different biological matrices such as feces (13, 28) or serum (24, 27). A similar situation occurs with PTB. MAP is a mycobacteria characterized by causing digestive disorders, making feasible to find these alterations directly reflected in the fecal volatilome. Despite of this, exhaled breath (1, 31, 32) and serum (30) have given promising findings in different animal species.

The rationale for analyzing exhaled breath in a model of chronic intestinal infection or feces in a primarily respiratory disease is based on the hypothesis that they do not only contain substances originated from the airways or from the digestive system. These also contain metabolites released *via* the lung or the intestine but originated and related to the whole metabolic or health state of the subject (1).

The three most used biological samples for VOC analysis of mycobacterial diseases in animals are exhaled breath, serum, and feces (Table 1).

### Exhaled Breath

The principle of using exhaled breath lies in its capability for discerning disease-related changes and biomarkers in the organism that are reflected into the breath through exchange *via* the lungs (25), because of its ability to cross the alveolar membranes before being exhaled (26). The use of exhaled breath offers several advantages because it is a non-invasive sample produced in ample supply, having the potential for direct, inexpensive, and eventually real-time monitoring (25, 90). Although in the literature it is considered as a sample that is relatively easy to obtain, its sampling methodology in animals is diverse, revealing a lack of standardization: from modified equine nebulization masks or nostril samplers for cattle, specific ventilators for mice or intubation for macaques, to automated alveolar sampling devices for goats. Furthermore, some factors can affect the sampling methodology, such as eructation in ruminants, which has been shown to significantly affect exhaled VOC profile (91). VOCs from breath are normally concentrated to sorbent materials, such as Tenax or Carbopack Y, Carbopack X, and Carboxen 1000 (18, 80), which simplify its transport and storage, and later these are used to quantify and evaluate the volatile substances with different analytical techniques.

Healthy and diseased animals have been successfully distinguished in mycobacterial infections by identifying volatile molecules in exhaled breath (Table 3). (18) performed breath collection and analysis in *M. bovis*–inoculated cattle with two strains obtaining good sensitivity and specificity: 83.8–96.4% and 97.4–99.2%, respectively, using the microbiological culture as reference technique. In addition, (25) reported the measurement of two VOCs from breath linked with *M. bovis* infection and other two VOCs associated with samples from negative individuals, obtaining sensitivity and specificity values of 100 and 79%, respectively.

The studies included in this revision evaluating exhaled breath in the context of mycobacteria infection highlight some important variables to be considered (41). The use of different animal species models, the *Mycobacterium* species and strain used, the infection phase, the breath volume collected, and the sorbent phases used to concentrate VOCs are factors that often differ between the existing assays. Considering all the aforementioned information, a comparison between the existing results is a challenge.

### Feces

Feces are regarded as the most accessible sample for research (92). Considering that feces constitute the main media for eliminating metabolic products, these are an important source of information about the internal homeostasis (17). The reason for testing changes in VOCs in feces is based on the common assumption that any abnormality in the activity or composition of the intestinal microbiota and in the whole organism may alter the odor of this matrix (1), which is supported by studies from both human (93–96) and animal medicine (97–100). Consequently, examination of volatile fecal emission could be a very useful non-invasive diagnostic approach (1).

However, as a remarkable fraction of VOCs found in feces is generated by gut commensal microbiota (101), a well-matched control group and knowledge on these bacteria are necessary to identify VOC patterns of pathogenic conditions (31). Despite this shortcoming, using feces as matrix has many advantages; besides an easier sampling, it is not necessary to restrain the animals, eliminating the stressful situation that it implies. Moreover, and in contrast with human medicine, feces offer many different possibilities in terms of sampling protocol: per rectum, after sacrifice, using laboratory animal cages, or just after defecation are some options in veterinary research. The studies using feces reviewed in the present work highlight the existing heterogeneity between the published results (Tables 1, 2). However, the obtained results have placed fecal volatilome analysis as an innovative diagnostic approach in the current research context for mycobacterial infections. In this sense, attention has been focused not only on the discrimination between infected and healthy individuals (1, 13, 31, 32) but also in the use of fecal VOC profile for other purposes, such as identification of vaccinated animals in white-tailed deer (13) and cattle (28).

### Serum

Serum is the sample of choice in many studies because of its relative ease to obtain and store, and safe distribution (24). Blood or serum is the means of transport of many different substances, compounds, and markers through the organism, existing a complex exchange with the lung or the intestine, among other systems (102). Alterations in VOCs from serum can be detected when a disease, an infection, or a pathologic condition occurs (103).

Serum has been used to distinguish the infection by *M. bovis* or MAP in different animal species through volatilome evaluation (Table 3) obtaining very interesting results. For example (30), were able to discriminate MAP and *Brucella* spp. infection in cattle through VOC analysis; and (27) reported an analyzing time of only 20 min to differentiate between bTB-infected and bTB-free bovine sera. However, and although blood and serum could be the most routine samples used in diagnostic field, its collection supposes a stressful situation as it is an invasive method that requires individual immobilization.

In conclusion, three different biological samples have been discussed as source of information in mycobacterial diagnosis in animals through volatilome analysis. Although interesting and useful findings have been shown, there is still a lack of homogeneity among many different study conditions. This often leads to non-comparable and inconsistent results. For example, despite studying the same pathogen (MAP), and using the same biological samples (exhaled breath and feces) and animal species (goats), contradictory conclusions can be found in the literature: while some showed that differences in VOC profiles were less pronounced from breath than those obtained in feces (31, 32), others suggest that volatilome evaluation from exhaled breath might be superior compared with the one from feces (1). In fact, the researchers usually acknowledge that their hypotheses should be verified by future studies, considering their findings as starting points (1, 32). Hence, no reliable comparisons or conclusions can be made with the available information, being advisable to carry out studies where the biological matrices are used simultaneously with the same methodological conditions.

## Instrumental Techniques

Some analytical instrumentation techniques allow VOC evaluation. Although gas chromatography with mass spectrometry (GC-MS) is referred very often as the “gold standard” for VOC analysis (104, 105), selected ion flow tube–mass spectrometry (SIFT-MS), proton transfer reaction mass spectrometry (PTR-MS), and secondary electrospray ionization mass spectrometry (SESI-MS) are other mass spectrometry–based options available for bacterial VOC analysis (40). Moreover, various types of ion mobility spectrometers (IMS), such as classical time of flight IMS (IMS-ToF), aspiration IMS (a-IMS), differential mobility spectrometers (DMS), field-asymmetric wave IMS (FAIMS), or multi-capillary column IMS-ToF (MCC-IMS-ToF) have been successfully used in identification of bacterial VOCs as well (22).

In the present review, different analytical techniques have been evaluated to assess VOCs as a diagnostic methodology for mycobacterial infection in animals (Table 3): different GC-MS modalities, various electronic nose (EN) models, and DMS, being the first two options by far the most frequent approaches. In this sense, as other researchers have previously indicated, the diverse methods of VOC collection and analytical systems that have been used are likely to have contributed to the results' variability (18). Supporting this context, each analytical method offers both advantages and limitations.

### Gas Chromatography Coupled to Mass Spectrometry

GC-MS has become one of the most preferred options for marker identification of bacterial origin with a very good sensitivity (40). It has a huge potential for both identification and quantification of unknown VOCs from complex matrices (106, 107). Ellis et al. (18) found that 4-hydroxy-4-methyl-2-pentanone, benzaldehyde, 1-ethyl-2-pyrrolidinone, α,α-dimethyl-benzenemethanol, and nonanal were present in significantly greater concentration in *M. bovis*–infected animals than in control ones. Moreover, Bergmann et al. (31) found 16 and 3 VOCs in feces and breath, respectively, which provide detectable differences at any infection time between MAP-inoculated and non-inoculated animals. GC-MS has the capacity of detecting VOCs within a range of parts per billion range, or lower, with good reproducibility and linearity (22, 25). In other words, GC-MS not only seems to be the most suitable for bacterial biomarkers search; in fact, it is the most used technique to diagnose these infections (40). The present review highlights the usefulness of GC-MS as an analytic tool to evaluate VOC changes due to mycobacteria infection employing different biological samples such as exhaled breath or feces (Table 3).

In spite of these many advantages, GC-MS has also several drawbacks: most GC-MS equipment are still not implemented as a portable tool; it requires high levels of expertise, qualified personnel, and pre-concentration techniques; and it is currently an expensive instrumentation (40). Therefore, given the aforementioned cons and the significant sampling and analysis time that it implies, GC-MS is not suitable for being used in end-user or point-of-care sites (25, 40).

It is also worth mentioning that comprehensive two-dimensional GC-MS (GCxGC-MS) stands out for the possibility of analyzing VOCs coming from complex matrices (40) and for providing a more complex and unparalleled separation as well as three-dimensional chromatograms' visualization (108).

### Electronic Nose

The electronic nose (EN) is an instrument based on chemical sensors combined with a pattern recognition system (109), able to detect different VOCs, such as odors, flavors, and vapors (110, 111). The main advantages of this methodology are the ease of use, its low price, and the rapid analysis time (27). Furthermore, EN methodology avoids sample transport to laboratory, positioning itself as one of the optimal techniques for pen-side use (27). However, it has problems with background separation, it does not identify substances detected, and sometimes its detection limit is high, giving insufficient sensitivity (31, 112).

The huge variety of applications where EN has shown effectiveness could be also considered as another of its strengths: versatility. In this sense, the reviewed information reveals the applicability of many types of EN sensors for different species of mycobacteria diagnosis through VOC identification (Table 3). Despite the good and interesting results obtained, the ease of transport of this device has not been exploited in depth because most studies using EN has analyzed VOCs from serum (Table 3) and not from other types of samples, such as feces or exhaled breath. The aforementioned information enhances the importance of carrying out future studies using EN focused on non-invasive biological matrices which would permit to develop a portable tool. In this sense (25), used their GC-MS results to tailor an artificial olfactory system to detect bTB in cattle exhaled breath. Although their new system successfully identified all infected animals (100% sensitivity), it wrongly classified 21% of the non-infected individuals (79% specificity).

### Other Minor Techniques

DMS is an IMS modality that has been occasionally used for volatilome assessment in mycobacterial infections (Table 3). This instrumentation has a lower cost, and it can be used alone or coupled with a GC column which acts as a pre-separation stage (40). Its relatively low price, robustness, reliability, and miniaturization turn IMS technology into one of the potential alternatives for portable VOC analysis in disease diagnosis (40). As with EN, one of its main drawbacks is its lack of capacity to identify specific VOCs (1). This analytical device used by (1) permitted to discriminate healthy from MAP-infected goats, noting a direct correlation among postmortem findings and *in vivo* measurements.

SIFT-MS is a quantitative technique for trace gas analysis based on the ionization of these volatile compounds by positive precursor ions along a flow tube. Although its main advantages are a rapid analysis time and a lower mass range, biological samples usually provide complex data which need computational assistance to be analyzed (24). Spooner et al. (24) applied multivariate analysis for the first time to SIFT-MS data to evaluate serum headspace analysis as a faster screening tool for *M. bovis* infection in badgers, obtaining a much faster diagnosis. However, the insufficient accuracy achieved (88% of true positive and 38% of false positive) makes this approach unsuitable as an alternative for conventional diagnostic techniques.

The existing differences between the reviewed analytical techniques suggest the importance of using methodologies, such as GC-MS, as a first-line analysis, with the objective to identify and define tentative biomarkers. Then, other approaches, such as IMS or EN, could be developed and adapted to a field or point-of-care use.

## Targeted Analysis vs. Non-Targeted Analysis

The diagnosis of an infection using VOC analysis can be reached by identifying specific substances related to the pathologic process or by detecting significant alterations in the whole VOC profile. Most of the research has attempted to isolate unique VOC biomarkers (targeted analysis) (Table 3) that would indicate the presence of mycobacterial infection, with little work done investigating potential changes within the whole VOC profile (non-targeted analysis).

There are VOCs that can be present in many different situations, hampering to find a specific substance for a particular infection or process. This is the case of methyl-nicotinate, a compound that, although it is proposed as a *M. tuberculosis* biomarker (28), can be found in the breath of non-tuberculous smokers (113); it is used as a flavoring ingredient, and it is present in coffee, various nuts, alcoholic beverages, and fruits (114, 115). In this sense, although tentative biomarkers have been associated with mycobacterial infection in both human (35, 37, 116, 117) and veterinary medicine (Table 3), the influence of different factors as well as the dynamic character of volatilome makes the identification of indicative or unique VOCs difficult (28). According to the literature, these factors may be related to host biological variables, environmental conditions, symbiotic and infectious microbe–host interactions, pathophysiological responses, the method of sample collection, and differences in analytical methods used for sample analysis (30, 85, 89). The bias induced by these factors is exemplified by the comparison of two studies which aimed to use exhaled breath VOCs as a source of information to diagnose *M. bovis* infection in cattle (18, 25); using the same animal species, pathogen, and biological sample, only two VOCs were consistent between both studies, highlighting the challenge that this approach supposes. However, along the present review, several VOCs have been pointed out due to its frequency and consistency between the included studies (Table 3); while nonanal, hexanal, 2-ethyl-1-hexanol, acetone, and 3-methylpentane were found to be present in both *M. bovis* and MAP infection, there were also compounds indicative of single infection. This is the case of indole, for *M. bovis*, and 2-butanone, methyl acetate, and 3-methylfuran for MAP, molecules that could be postulated as candidates for the discrimination between MTBC and MAP processes. The aforementioned VOCs were found to be consistent between different studies (13, 18, 28, 29, 31, 32), matrices (feces and exhaled breath), and animal species (cattle, goat, deer, and wild boar), which opens up a huge opportunity to use this approach as a diagnostic option for general mycobacterial infections and specific infections as well. Nonetheless, there is still no specific biomarker for mycobacterial infections, being priority to develop analytical methods adapted to volatilome characteristics which allow adequate identification and quantification of these molecules.

On the other hand, there are already studies in the literature which have used the entire VOC profile (non-targeted analysis) to successfully discriminate between diseased and non-infected animals (28). In this way, many research groups have highlighted the importance of considering the entire profile of VOCs released by specific pathogens and how these profiles can help discriminating between infecting pathogens, rather than relying on a limited number of biomarkers (targeted analysis) (118). However, non-targeted analysis does not identify compounds, making not feasible to gather information about the source of these compounds. In addition, other factors, such as feeding, environmental conditions, or metabolic variables, need to be fixed to draw conclusions from the results obtained using this methodology. In this sense, non-targeted analyses by EN or DMS (1, 23, 27, 30), although showing volatilome potential, make difficult the comparison with other studies, underlining the importance of a proper VOC identification and quantification to obtain consistent results.

## Concluding Remarks and Future Prospects

In conclusion, although currently there is an important research trend that evidences the potential of VOCs emitted in mycobacterial infections in animals as a diagnostic tool, it is still in an initial phase and presents some difficulties. The number of *in vivo* assays which study the implementation of the analysis of VOCs for mycobacterial diagnosis in animal research is considered scarce. Furthermore, considering the lack of standardization, the dynamic nature of volatilome, the drawbacks and differences in the current methodology, and the use of biological matrices, inconsistent and non-comparable results are usually obtained. Thus, no singular biomarkers indicative of mycobacterial infections have been described to date. The high number of research groups that have studied this new approach worldwide contributes to the lack of standardization because they usually use different protocols, a reason that makes more difficult to reproduce their results.

In the authors' view, volatilome analysis is considered an innovative approach which is likely to become of interest as a complementary tool for current diagnostic methods; this approach is not presented as an alternative, at least to date, but it is considered a strategy that could offer significant and complementary advances. Although the strategies based on IST and serology have partly succeeded for control and eradication campaigns of MTBC and MAP, respectively, volatilome features could allow the development of an *ante-mortem*, portable, and non-invasive technique, possibly used as a field screening method able to improve sensitivity and specificity parameters as the collected data highlight (Table 2). In addition, the possibility of discrimination of highly related mycobacteria infections and the detection of infected subclinical animals stand as major ambitions. Further and thorough studies using several biological matrices with constant *in vivo* conditions are required to obtain robust results as well as reliable comparisons and check the consistency of this methodology between different assays before its implementation at field level. Against the previously described background, the development of analytical tools to obtain useful and robust information about potential VOC marker identification and quantification is considered of paramount importance. This will open new and complementary possibilities in the questioned diagnosis of mycobacterial infection and help to overcome the described drawbacks in the present revision.

## Author Contributions

JG-L and PR-H conceived and designed the review. PR-H analyzed the data and wrote the manuscript. JG-L, VR-E, and LA revised the manuscript. All authors read and approved the final manuscript.

## Conflict of Interest

The authors declare that the research was conducted in the absence of any commercial or financial relationships that could be construed as a potential conflict of interest.
